# Advances in Metagenomics and Its Application in Environmental Microorganisms

**DOI:** 10.3389/fmicb.2021.766364

**Published:** 2021-12-17

**Authors:** Lu Zhang, FengXin Chen, Zhan Zeng, Mengjiao Xu, Fangfang Sun, Liu Yang, Xiaoyue Bi, Yanjie Lin, YuanJiao Gao, HongXiao Hao, Wei Yi, Minghui Li, Yao Xie

**Affiliations:** ^1^Department of Hepatology Division 2, Beijing Ditan Hospital, Capital Medical University, Beijing, China; ^2^Department of Hepatology Division 2, Peking University Ditan Teaching Hospital, Beijing, China; ^3^Department of Gynecology and Obstetrics, Beijing Ditan Hospital, Capital Medical University, Beijing, China

**Keywords:** metagenomics, shotgun sequencing, next-generation sequencing, third generation sequencing, antibiotics resistance genes, bioremediation

## Abstract

Metagenomics is a new approach to study microorganisms obtained from a specific environment by functional gene screening or sequencing analysis. Metagenomics studies focus on microbial diversity, community constitute, genetic and evolutionary relationships, functional activities, and interactions and relationships with the environment. Sequencing technologies have evolved from shotgun sequencing to high-throughput, next-generation sequencing (NGS), and third-generation sequencing (TGS). NGS and TGS have shown the advantage of rapid detection of pathogenic microorganisms. With the help of new algorithms, we can better perform the taxonomic profiling and gene prediction of microbial species. Functional metagenomics is helpful to screen new bioactive substances and new functional genes from microorganisms and microbial metabolites. In this article, basic steps, classification, and applications of metagenomics are reviewed.

## Introduction

Traditionally, research on microorganisms is based on cultured organisms, which has several disadvantages (Kellenberger, 2001). In 1998, Metagenome, also known as microbial environmental genome (Handelsman et al., 1998), is defined as “the genome of the total microbiota found in nature.” Metagenome currently refers to the sum of the genomes of bacteria and fungi in environmental samples. Metagenomics is the study of a collection of genetic material (genomes) from a mixed community of organisms. In short, metagenomics is a new way to study microorganisms in a specific environment by using functional gene screening or sequencing analysis. The main areas of concern of metagenomics research are microbial diversity, population structure, genetic and evolutionary relationships, functional activity, and cooperative relationships, and relationship with the environment. Metagenomics research is developing rapidly in medicine, agriculture, environmental protection, and other fields.

This article reviews the development and application of metagenomics detection technology. The review is divided into three parts. The first part focuses on molecular biology technology from the extraction of all microbial genes, heterologous expression of gene fragments, to the screening of target genes or active products, and finally the sequencing of target genes. The second part introduces the main classification or branch of metagenomics from the research purpose or objects. The third part is about the application of metagenomics in various fields.

## Basic Steps of Metagenomics

Metagenomics is based on gene cloning. The basic procedure of metagenomics consists of the following steps. First, all genes in environmental microbial samples are extracted and enriched. Second, the genes are cloned into the vector, which is transformed into host bacteria to establish the metagenomic library. Finally, the metagenomic library is screened and analyzed. The extraction of metagenomic DNA and construction and screening of metagenomic library are essential.

### Extraction and Enrichment of Metagenomic DNA

The traditional approach is to culture microorganisms and then extract the DNA, but 99% of the microorganisms in the environment cannot be cultured (Torsvik et al., 1990; Handelsman et al., 1998). Extracting high concentration and large fragment environmental microbial total DNA is the first and most critical step in building a metagenomic library. There are two key points to extracting metagenomic DNA. First, to extract all the genes of all microorganisms in the sample. Second, to keep the integrity and purity of the fragment. Sample collection must follow a strict procedure to extract as much DNA as possible from environmental microorganisms and maintain large DNA fragments.

According to whether or not the cells are isolated before DNA extracting, it can be divided into direct extraction and indirect extraction. Direct extraction uses physical (such as freeze-thaw) and chemical (such as the addition of protease or SDS) to lyse microorganisms directly to release microbial DNA (Gabor et al., 2003). The total DNA in all samples can be obtained by the direct extraction method, which is suitable for extracting 1–50 kb DNA. The direct extraction method is simple and efficient, but the purity is low (Schneegurt et al., 2003; Wang et al., 2011). The indirect extraction method requires isolating the microbial cells at first and then extracting DNA using a gentle method. The DNA obtained by indirect extraction is of high purity and suitable for the extraction of 20–500 kb DNA. The indirect extraction method is cumbersome and inefficient, which may cause the loss of some microbial DNA (Gabor et al., 2003).

The sample size depends on microbial concentration. The high density of microorganisms such as stool samples may require only an anal swab sample. To study the marine microbial community, which is a low-density microorganism, large numbers of water samples are captured and concentrated through filters. Samples need to be decontaminated. For example, humic acid in soil is often tightly bound to DNA, so humic acid needs to be removed during sample preparation (Daniel, 2005). When studying microorganisms in humans or animals, the DNA contamination in samples and host must be removed.

For DNA samples with low abundance, enrichment at the cell level and gene level can be carried out after DNA fragment extraction, but this may cause certain deviations (Probst et al., 2015). The common methods of gene enrichment are stable isotope probing (SIP) (Chen and Murrell, 2010), suppression subtractive hybridization (SSH) (Galbraith et al., 2008; Chew and Holmes, 2009), DNA chip technology, etc. (Avarre et al., 2007).

### Construct Metagenome Libraries

In the construction of a metagenome library, microbial DNA should first be cut mechanically or digested into a certain size of DNA, and then randomly recombined with an appropriate clone vector, and finally transformed into suitable host cells. The goal is to isolate some target genes or preserve the entire DNA of an organism. The strategy of building microorganism DNA library changes with the research objective: low abundance genes or high abundance genes and small segments of a single gene or large segments of gene clusters in a metabolic pathway.

The vector is selected only if it is conducive to the amplification of the target DNA fragment and is convenient for screening after the transformation and expression. The common vectors include plasmid, cosmid, fosmid, or bacterial artificial chromosome (BAC), yeast artificial chromosome (YAC), and phage. Plasmids are suitable for cloning DNA fragments below 10 kb. Cosmid and fosmid are suitable for cloning large genes or multiple gene fragments (20–40 kb). BAC and YAC can be used to clone DNA fragments of 200–450 kb. The BAC shuttle vector, which can be duplicated and expressed in a variety of hosts, can be used to expand the host range (Kakirde et al., 2011).

The host cells are generally the ones with good stability of the recombinant vector, high efficiency of transformation, and effective expression of the target gene fragment. Usually, different host strains are selected according to different research purposes. *Escherichia coli* is easy to culture and is the most common host, but it is a prokaryotic host with limitations. Some new host cells are gradually applied, such as host *Streptomyces* (Rebets et al., 2017), *pseudomonas* (Craig et al., 2010), and *mycobacterium*.

There are many barriers to the study of foreign gene expression in metagenomics. For example, the target gene may be lost in the process of library construction, and the host cell cannot recognize the unique promoter, failing the expression of heterologous gene fragments. To solve this problem, multi-host expressions can be adopted. A research group expressed DNA fragments in 6 different bacteria (Craig et al., 2010). If the target gene is in cluster form, large segments of DNA need to be inserted to obtain complete metabolic enzymes and pathways. At present, the failure of DNA cloning in large fragments may be caused by the destructive effects of physical and chemical actions during the extraction process or the limited receptive capability of host bacteria.

### Screening of Metagenome Library

Currently, there are three approaches to environmental metagenomic library screening: function-driven screening, sequence-driven screening, and substrate-induced gene expression screening (SIGEX). Other techniques include DNA stable-isotope probing (DNA-SIP) (Chen and Murrell, 2010) and fluorescence *in situ* hybridization.

There are two kinds of functional screening. One is to screen the expression of active products of exogenous genes in host cells according to special characterization (such as color and plaque) on various selective media to obtain target clones (Apolinar-Hernández et al., 2016; Popovic et al., 2017). The other is to screen the host strain and its mutants based on their complementary growth characteristics under selective conditions (Cheng et al., 2017). The function-driven screening method is fast, simple, and does not rely on any known sequence information. Most biocatalysts, such as protease (Apolinar-Hernández et al., 2016), esterase (Popovic et al., 2017), are obtained by this method. The biggest disadvantage is that it depends on the expression of functional genes in foreign hosts, so the success rate of screening is very low. Thus, selecting the right host and cloning the full length of a gene or gene cluster is required. Functional analysis is suitable for gene screening of large fragment DNA libraries.

Sequence analysis usually uses oligonucleotide primers or probes to screen target genes by molecular biological methods such as PCR and gene hybridization. Only target clones with known sequences can be screened. Sequence analysis is suitable for screening highly conserved sequence enzyme genes, such as polyketide synthase gene (Ginolhac et al., 2004) and cellulase genes (Voget et al., 2003). Sequence analysis is also suitable for gene screening of small DNA fragments. The advantage of sequence analysis is that it need not rely on host cells to express the cloned genes, but the disadvantage is that it is unable to screen the unknown genes with completely different sequences from the existing genes.

Some new methods such as microarray, stable isotope labeling, and fluorescence *in situ* hybridization can also be applied to sequence screening. The DNA microarray technology, also known as gene chip technology, is based on nucleic acid hybridization (Avarre et al., 2007). First, DNA fragments are densely and orderly arranged on the solid support such as silicon wafer to form DNA microarray. Then the labeled samples are hybridized with the DNA microarray, and the genes are analyzed according to the hybridization sites and signal strength. DNA microarray technology can be used to study key genes in metabolic pathways. The DNA microarray technology has advantages of rapid detection and preliminary screening, but its specificity and sensitivity are only 1/100–1/10,000 of PCR method, so it is unsuitable for the detection of unknown functional genes (Call, 2005; Palka-Santini et al., 2009; Mauk et al., 2015).

The principle of substrate-induced gene expression screening is that metabolism-related genes or enzyme genes are usually expressed in the presence of substrate-induced conditions (Uchiyama et al., 2005; Yun and Ryu, 2005). SIGEX has several advantages, such as no modification of the substrate is required and functions of unknown enzymes and genes can be inferred from the substrate. SIGEX is a high-throughput screening method that is suitable for industrial use. But it is highly demanding on the structure and fitness of the target gene in the host and is only suitable for substrates that can enter the cytoplasm and target gene, which itself has a promoter.

What metagenomics need now are high-throughput screening methods. In 2015, a research team attempted to apply microfluidic chips integrating sample detection and automatic analysis (Colin et al., 2015), which is known as lab-on-chip (LOC). LOC only requires a sample of nanoliters or picoliter level, but the technical requirements are high. LOC requires advanced technologies such as chip sampling technology, microfluidic technology, and focusing electrophoresis technology. LOC implements various experimental steps in the laboratory, such as sample preparation, biochemical reaction, and sample detection, on a chip of several square centimeters. LOC meets the objective of overall miniaturization, automation, integration, and portability of sample testing.

### Metagenomic Sequencing and Analysis Techniques

Rapid progress has been made in sequencing technologies, from shotgun sequencing to high-throughput, next-generation sequencing (NGS) (Mardis, 2008; van Dijk et al., 2014), and third-generation sequencing (TGS) (Branton et al., 2008).

#### Preparation of Metagenomic Sequencing Samples

Metagenomic DNA can be sequenced from DNA libraries obtained from target gene cloning or directly from samples. The purity and content of the DNA must be ensured before it is sequenced.

#### History of DNA Sequencing Technologies and Platforms

**Figure 1:**
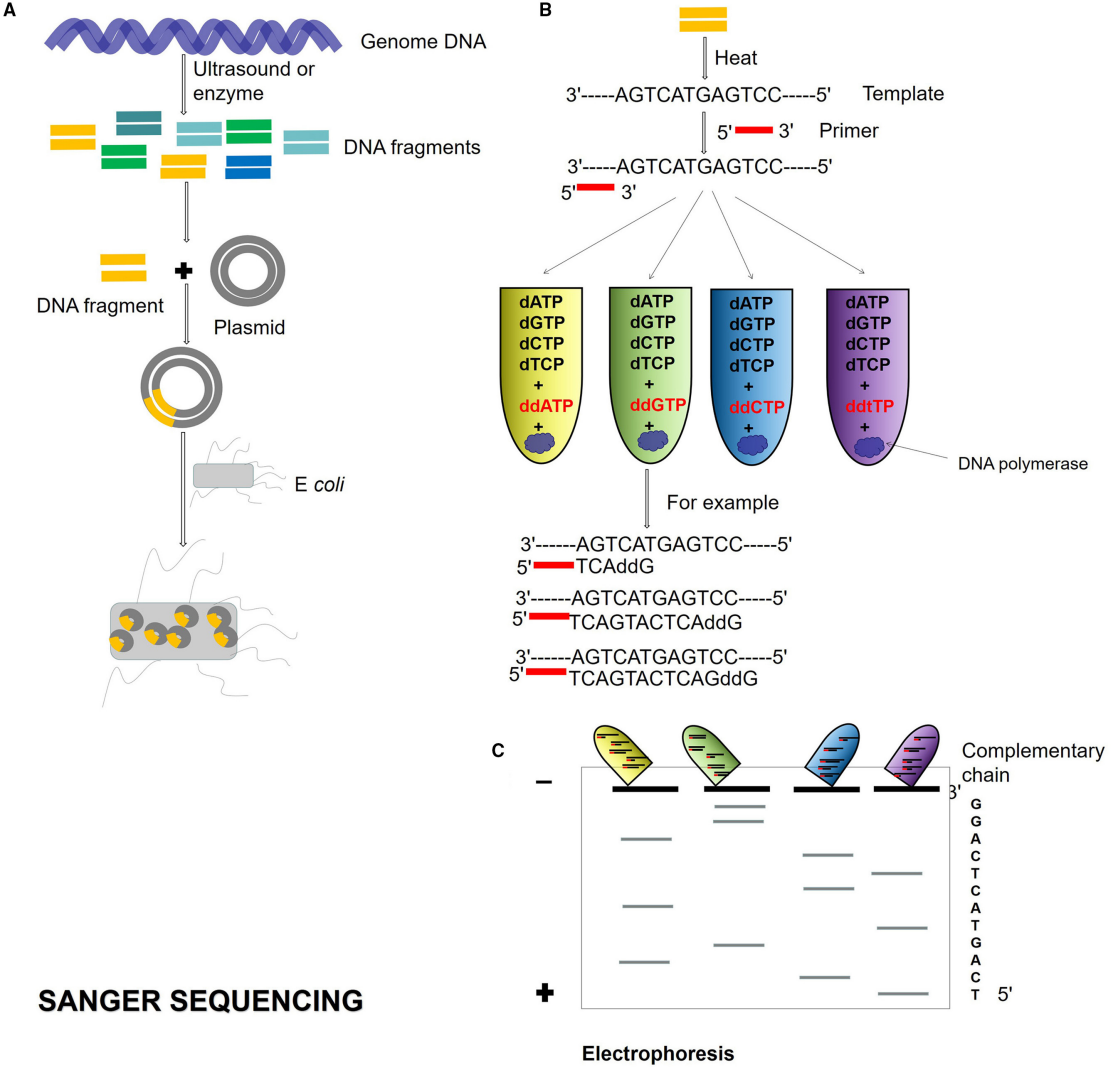
Sanger sequencing process. **(A)** Genome DNA is cut into different DNA fragments by ultrasound or enzyme, connected into a plasmid, and transformed into *Escherichia coli*. **(B)** The Dideoxy chain termination method is used for sequencing. The reaction system is a mixture of template, primer, DNA polymerase, and dNTPs. Four kinds of ddNTP (ddATP, ddTTP, ddCTP, and ddGTP) with radioisotope marks are, respectively, added to four reaction systems. If ddNTP is incorporated into the oligonucleotide chain, the 2′ and 3′ of ddNTP do not contain hydroxyl groups and cannot form phosphodiester bonds, then the extension of the DNA chain is terminated. **(C)** Four PCR reaction system products are added to gel electrophoresis, and autoradiography was used to determine the DNA sequence according to the location of electrophoresis.

Second-generation DNA sequencing was established on PCR techniques. The first-generation sequencing is the shotgun sequencing based on the traditional Sanger sequencing technology (Olson, 1993; Bankier, 2001; Quince et al., 2017; Figure 1), and the sequencing using the dideoxy chain termination method is the gold standard of sequencing. The accuracy of shotgun sequencing is higher than those of second and TGS. It has a high accuracy of long reads from 700 to 1,000 bp and can deal with repeated sequences well. The disadvantage is that only one template can be detected at a time, which is slow and time-consuming.

**Figure 2:**
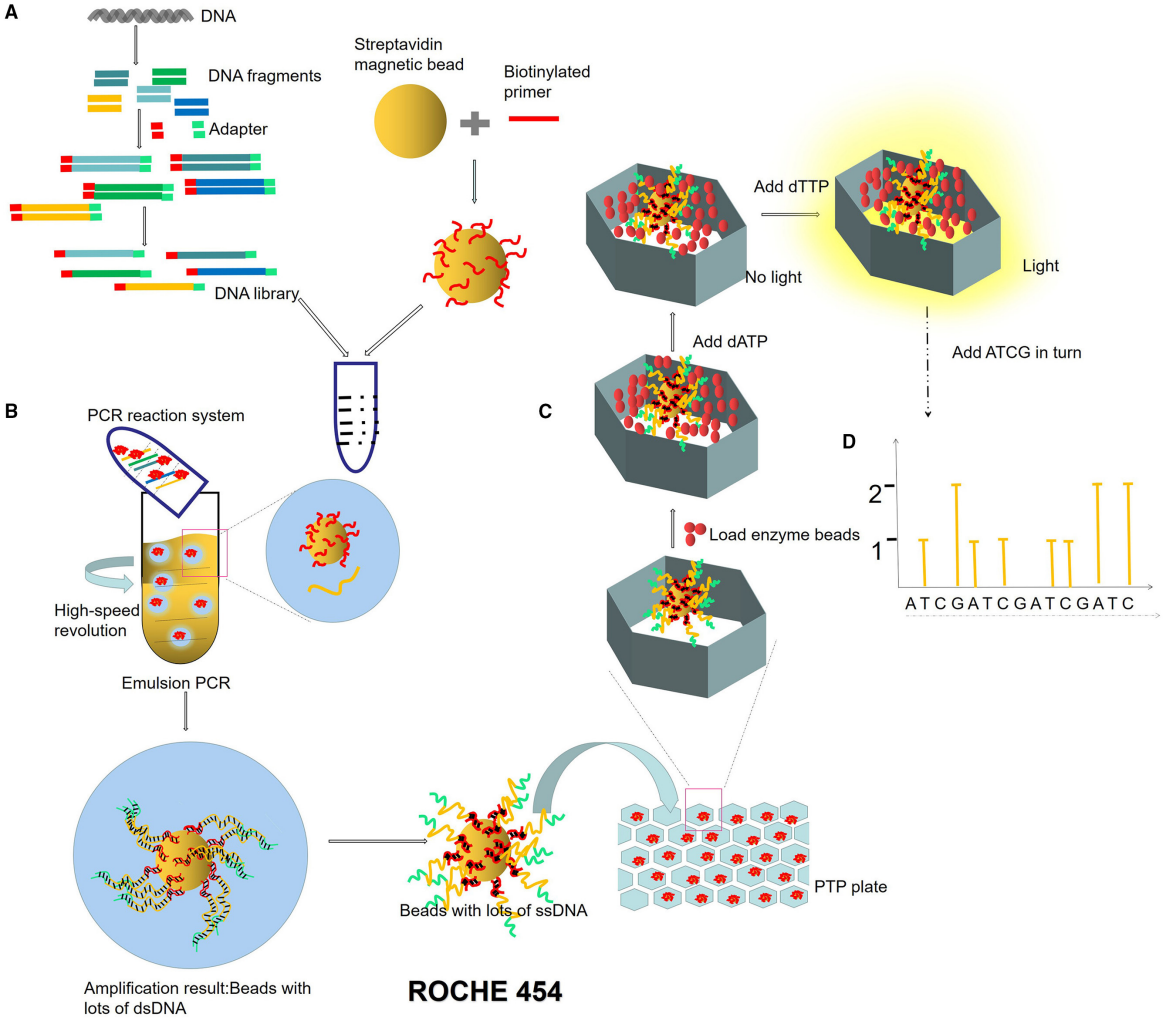
ROCHE 454 sequencing process. **(A)** Construction of micro-reaction system. DNA library is constructed by adding an adapter at the ends of DNA fragments. The magnetic beads combined with primers are mixed in the PCR reaction system (DNA fragments, enzymes, dNTPs). **(B)** Emulsion PCR. PCR microreaction systems are formed by injecting water (PCR mixture) into the oil, and each system contained only one template and one bead. **(C)** Pyrophosphate sequencing. Pyrophosphate technology is an enzyme cascade chemiluminescence reaction in the same reaction system catalyzed by four enzymes. In each circle of the sequencing reaction, only one dNTP is added. If it just matches the next base of the DNA template, it will be added to the 3′ end of the sequencing primer under the action of DNA polymerase and release a molecular PPi at the same time. Under the action of ATP sulfurylase, luciferase, and apyrase, an enzyme cascade chemiluminescence reaction is triggered by the PPi. **(D)** Light signal reception.

**Figure 3:**
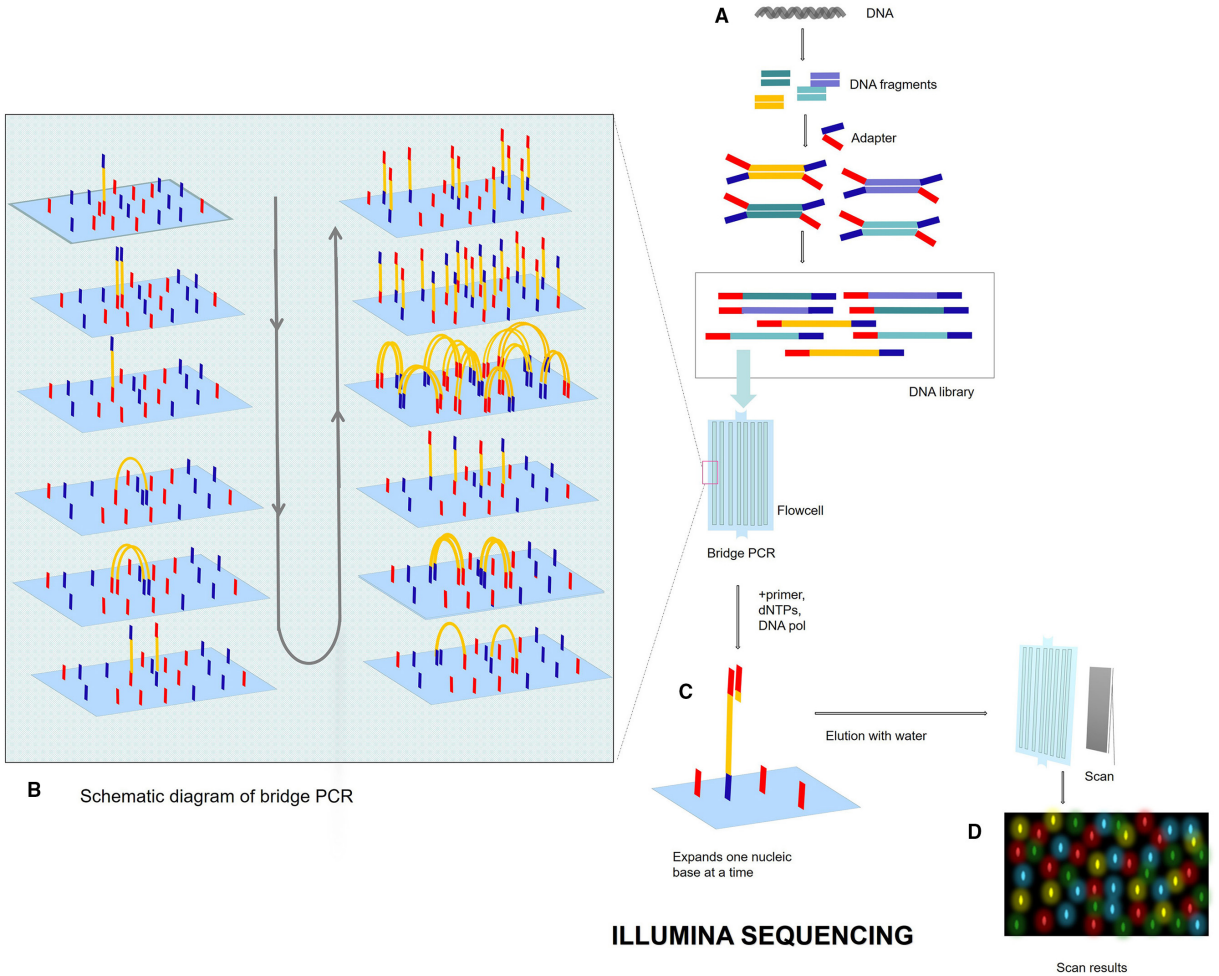
Illumina sequencing process. **(A)** DNA library. Breaks the genome DNA to form DNA fragments, adds adapters at both ends, and constructs a single-stranded DNA (ssDNA) library. After adding DNA library into flowcell, DNA fragments will be attached to the surface of flowcell when passing through flowcell. **(B)** Bridge PCR. Each DNA fragment is clustered in its position. After bridge amplification, each cluster contains many copies of the ssDNA template. **(C)** SBS: DNA polymerase, primers, and four dNTPs with specific fluorescence are added to the reaction system at the same time. The 3′ end of these dNTPs is connected with an azide group, which can block the incorporation of the next base, so only one base can be extended at a time. After washing off the remaining dNTP and enzyme with water, photos are scanned. After scanning, add chemical reagent, cut off the azide group and quenching fluorescence, and next cycle. **(D)** Fluorescent signal reception.

**Figure 4:**
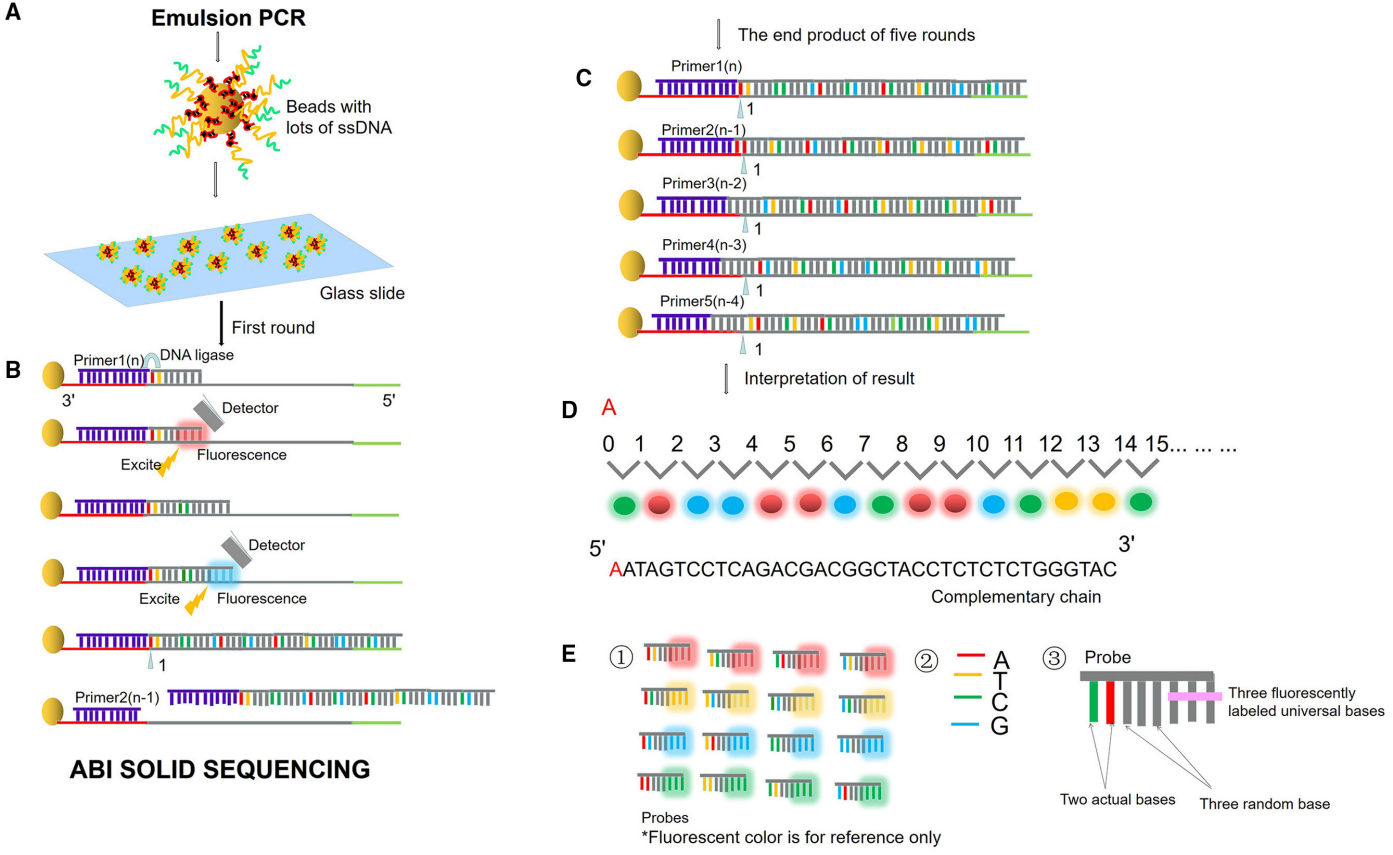
ABI sequencing by oligonucleotide ligation and detection (SOLiD). **(A)** Emulsion PCR. After the emulsion is amplified, a large number of amplified products from the DNA template are attached to the magnetic beads. Then, the magnetic beads are deposited on the glass slide. **(B)** SLB. The first circle of sequencing reaction includes: the sequencing primer is combined with the template; the probe is combined with the template; the ligase connects the sequencing primer and the probe, excites the probe fluorescence, and removes the last three bases of the probe. Then plus the second probe, connects the two probes with the ligase, excites the probe fluorescence, and removes the last three bases of the probe. Repeat the above reaction to synthesize the whole chain. **(C)** Solid template shift. After cutting off the whole synthetic ligation, add sequencing primer n-1, repeat the above steps and start the second circle of reaction. Unidirectional solid sequencing requires five circles of sequencing reactions. The primers are n, n-1, n-2, n-3, and n-4, respectively, and the bases at each position are detected two times. **(D)** Fluorescent signal reception and interpretation of results. **(E)** Two-base encoding principle. ① “two-base encoding” specifies the corresponding relationship between different base pairs and four probe colors in the coding region (e.g., red fluorescence represents AT, TC, CA, GT). ② Four bases. ③ The probe is an 8-base single-chain fluorescent probe. The base pairs at positions first and second of the probe are determined, positions from third to fifth are random bases, which can be any of the four bases of “A, T, C, and G,” and positions from sixth to eighth mark four fluorescent dyes of different colors.

**Figure 5:**
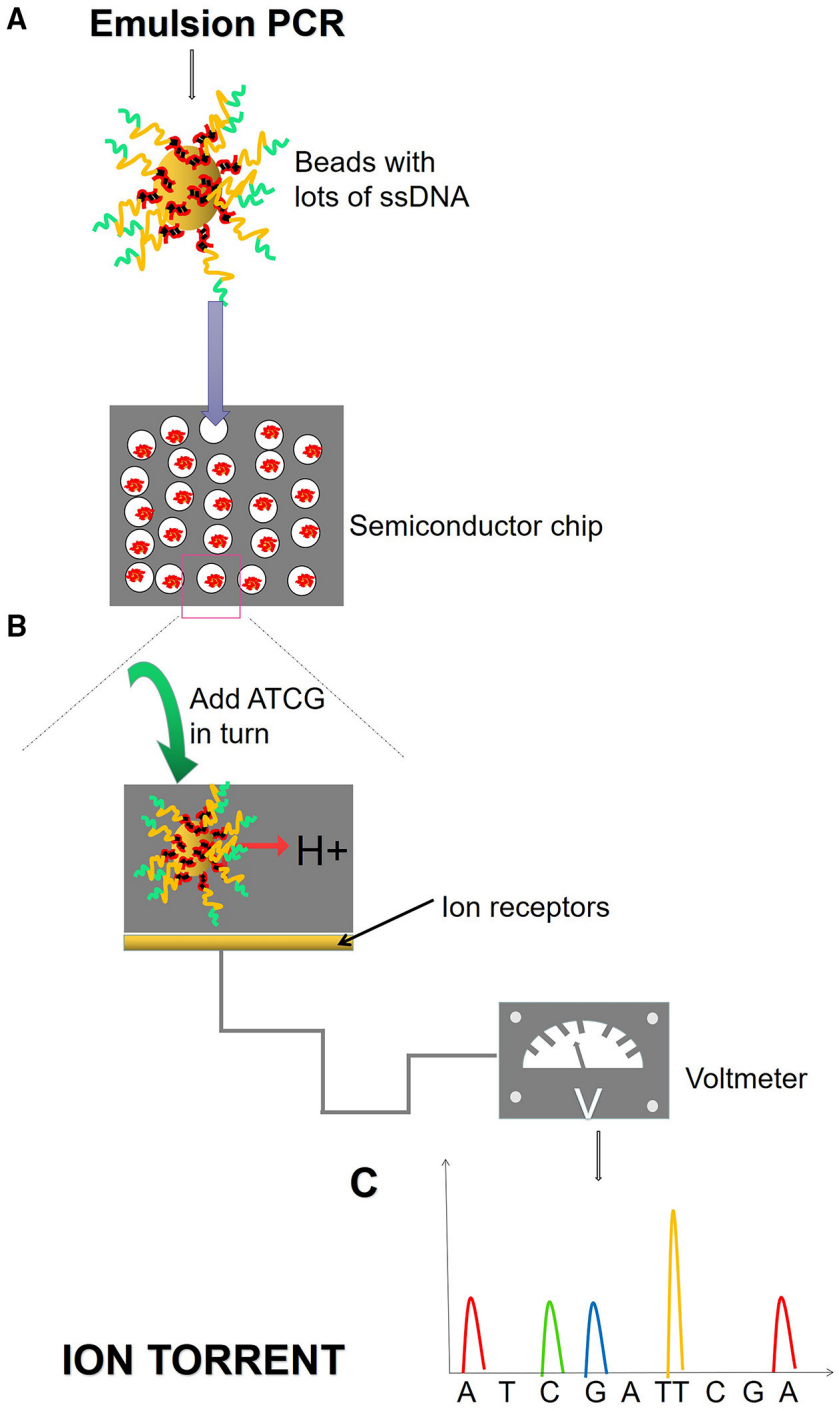
Ion torrent process. **(A)** Emulsion PCR. After the emulsion is amplified, a large number of amplified products from the DNA template are attached to the magnetic beads. Magnetic beads are fixed in the micropores of the semiconductor chip, and each micropore can only contain one magnetic bead. **(B)** SBS. Fix the ssDNA in the micropore of the semiconductor chip and add different dNTPs in turn. When the DNA polymerase polymerizes the nucleotide onto the DNA strand in the extension, it will release an H^+^, causing the change of pH value and voltage in the reaction system. **(C)** The electrical signal is accepted and converted into base information.

Next-generation sequencing uses large-scale parallel sequencing technology, which can simultaneously synthesize millions of complementary chains of sequencing templates and acquire sequence data. High-throughput and low-cost NGS is used worldwide in large-scale genome sequencing and resequencing. The NGS mainly used sequencing by synthesis (SBS) technology and sequence by ligation (SBL). Roche has developed GS FLX and GS Junior sequencing platforms with 454 sequencing technology and SBS (Marsh, 2007; Harrington et al., 2013; Figure 2), which can read more than 400 bp. Illumina used SBS and a 3′ blocked reversible terminator for sequencing (Shendure and Ji, 2008; Ansorge, 2009; Figure 3). Based on this technology, the Genome Analyzer platform reads 125 bp. Illumina sequencing has become the mainstream product of NGS. Applied Biosystems, InC. (ABI) used SBL to develop the ABI Solid Sequencer (Mardis, 2008; Shendure and Ji, 2008; Ansorge, 2009). The advantage of sequencing by oligonucleotide ligation and detection (SOLiD) (Figure 4) is that each base can be detected two times by two-base encoding, and the detection accuracy is higher than the other NGS. The disadvantage is that only 40 bp can be read, which is only suitable for SNP mutation detection. In addition, ion torrent sequencing technology developed by Life Technologies/Thermo Fisher Scientific adopts semiconductor chip technology (Figure 5). Four kinds of dNTPs sequential flow through the chip in turn, if one dNTP is complementary to the DNA molecule in a specific microhole of the chip, the dNTP is synthesized into the DNA strand. Subsequently, the hydrogen ions are released as the DNA strand stretches, causing the change of the pH. Finally, voltage changes are caused. Ion Torrent has the advantages of low cost, only an electrical signal detection system, and no light/fluorescence detection system (Pennisi, 2010).

Third-generation sequencing also called *de novo* sequencing technology, is a milestone in sequencing technology, which is based on single-molecule sequencing technology (SMS) (Xu et al., 2009) and large-scale parallel sequencing technology. There are two methods for the TGS. The first is single-molecule fluorescence sequencing. The representative technology is the American Helicos SMS (True single molecular sequencing, TSMS) and single molecular real-time sequencing (SMRT) technology from Pacific Bioscience (Xu et al., 2009; Rhoads and Au, 2015; Midha et al., 2019). SMRT Principle is SBS. DNA polymerase is anchored to the nanopore. The four fluorescent-labeled dNTPs emit different light in the base-pairing stage. The type of entering base can be determined according to the light wavelength and peak value. The phosphate group of dNTP is labeled with fluorescence in SMRT. The long reads are determined by DNA polymerase activity. SMRT requires a high-efficient optical detection system.

**Figure 6:**
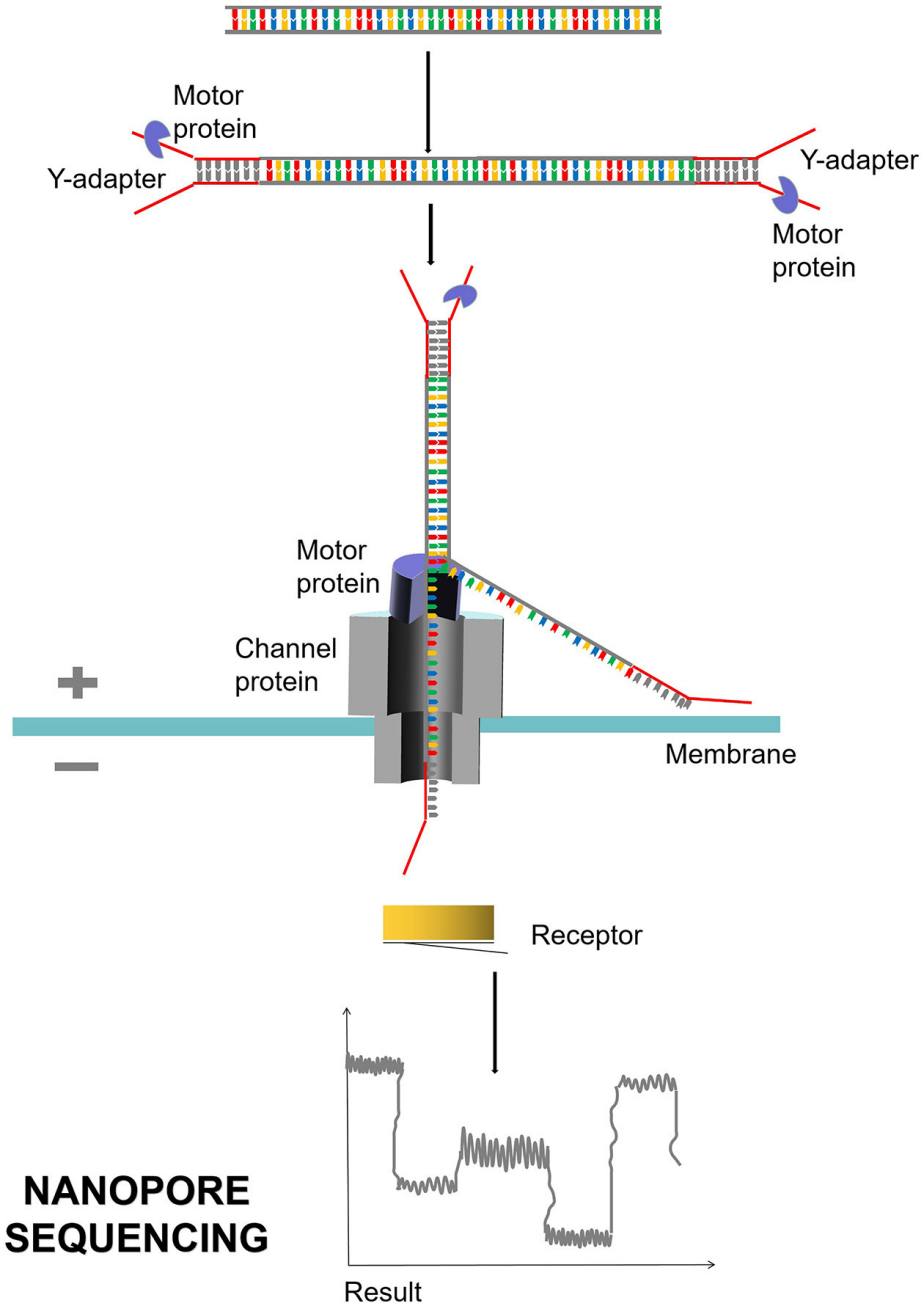
Nanopore sequencing process. Nanopore sequencing mainly uses nanopore protein, also known as reader protein, which is a transmembrane channel protein. Different voltages are applied on both sides of the membrane. DNA double strand is decoiled under the traction of motor protein, and a single molecule is driven by electrophoresis to pass through reader protein one by one. Different electrical signals are produced and detected when the bases of ATCG pass through.

The second is nanopore sequencing, which is based on electrical signals and single-molecule long-read detection. The representative company is Oxford Nanopore (Branton et al., 2008; Midha et al., 2019; Figure 6). The principle of nanopore sequencing is to use electrophoresis technology to drive individual molecules through nanopores one by one for sequencing. The electrification properties of each ATCG base are different during nanopore sequencing, and the difference of electrical signals detected can correspond to different bases. Compared with NGS, TGS is single-molecule sequencing. TGS has no PCR amplification preference or GC preference and can be sequenced directly without PCR amplification. The TGS has a very long read length, with an average read length of 10–15 kb and a maximum read length up to 40 kb. TGS can directly detect DNA methylation. The disadvantages of TGS are mainly shown in the following three aspects. First, sequencing errors occur randomly, with error rates of each base as high as 15%, which can be overcome and corrected by multiple sequences. Second, the cost of sequencing is high. Third, SMRT sequencing technology requires enzymes, and currently, better enzymes with much more activity and stability are needed.

When selecting different sequencing methods, there are many factors to consider, including the length of the target gene, the requirement of sequencing accuracy, the purpose of sequencing (whole genome sequencing, resequencing, or SNP), and the gene abundance of the sample. The sequencing platform should consider factors such as sequencing speed, read length, cost, error rate, methylation, and others. At present, NGS is the mainstream sequencing method, but TGS is still in the initial stage of development due to its high cost and high error rate in a single base. In practical applications, the advantages in long reads of 10–15 kb with low assembly difficulty of TGS and the characteristics of high quality in short reads of NGS can be utilized (Kumar et al., 2019; Table 1).

**Table 1:** Performance COMPARISON of seven sequencing instruments.

**Platform**	**Sequencing rules**	**Advantages**	**Disadvantages**	**Signal received**
Shotgun sequencing	Dideoxy chain termination method, capillary electrophoresis	Accuracy 99.999%, Reads 1,000 bp, High accuracy of processing repeated base, *De novo*	Low-throughput, high cost	Fluorescence
Roche 454	Pyrophosphate sequencing, SBS, Emulsion PCR	Accuracy 99%, Reads 400–600 bp, Parallel sequencing	Base insertion and deletion errors	Light
Illumina	SBS, Bridge PCR, Reversible terminator	Accuracy 99%, Reads 75–300 bp, Parallel sequencing	Base substitution error	Fluorescence
ABI solid	SBL, Emulsion PCR, Solid phase template shift, Two base encoding	Accuracy 99.94%, Reads 40 bp, Parallel sequencing, each base for sequencing twice	Prone to continuous base interpretation errors	Fluorescence
ABI/Ion torrent	Semiconductor sequencing, SBS, Emulsion PCR	Accuracy 99%, reads 200 bp, Parallel sequencing No light/fluorescence detection system	Difficult to identify homopolymers >8 bases, Base insertion and deletion errors	Electrical signal
Pacific bioscience	SMRT, SBS	Sequencing without PCR Detecting methylated bases average reads 10–15 kb	Random errors (5–15%) for a base, repeated sequencing can correct random errors	Fluorescence
Oxford nanopore	Nanopore sequencing Single-molecule sequencing, Direct detection of bases	Sequencing without PCR, No light/fluorescence detection system, Detecting methylated bases average reads 10–15 kb, longest to 40 kb	Random errors (5–15%) for a base, Repeated sequencing can correct random errors	Electrical signal

*SBS, sequencing by synthesis; SLB, sequence by ligation; ssDNA, single-stranded DNA; SMRT, single molecular real time sequencing. Solid (sequencing by oligonucleotide Ligation and Detection)*.

Different sequencing methods have their advantages and disadvantages. Shotgun sequencing is still in use today and serves as the gold standard because of its high accuracy. NGS has parallel sequencing after PCR amplification of the template and shows high throughput. At present, the market share of NGS is the highest. Illumina is currently the most widely used platform in the NGS. The Roche 454 sequencing technology has been withdrawn from the market due to its high cost. The TGS sequence directly, and the read length is longer, but the error rate is higher. Therefore, below are some suggestions for sequencing method selection. (1) For SNP detection of genome or insertion DNA sequencing of positive clones, shotgun sequencing is recommended. (2) If whole genome sequencing is carried out, Illumina is a better choice among NGS. (3) If cost is not considered, the combination of NGS and TGS is the best choice, which has high-quality short reads of NGS to correct wrong bases generated by TGS. The future direction of sequencing is to expand the microbial reference gene database and increase the accuracy of long reads.

#### Processing of DNA Sequencing Information Data

After a large number of reads are obtained by preliminary metagenomic DNA sequencing, the reads should be assembled into a complete genome sequence by software, and taxonomic profiling should be obtained at the same time. Then gene prediction and function annotation are carried out.

Genome sequencing mainly includes quality control (QC), sequence assembly, sequence binning, taxonomic profiling, gene prediction, and function annotation.

Quality control mainly includes two parts: de-noising and de-host sequence. It is necessary to establish a database of host sequences to be used for comparing and removing by sequence alignment (Schmieder and Edwards, 2011; Hong et al., 2014). Data analysis includes assembly-based and assembly-free metagenomic profiling. If the microbial genetic information database is complete, the post-QC data can be directly analyzed without assembly. There are currently two approaches to sequence assembly, relying on reference sequence assembly and *de novo* assembly (Iqbal et al., 2012; Quince et al., 2017; Breitwieser et al., 2019; Olson et al., 2019; Anyansi et al., 2020). Binning refers to the sorting and clustering of metagenome sequenced segments by species. Binning operations may result in bins of unknown microbial genome sequences that cannot be cultured in the laboratory. Binning can be divided according to different types of aggregation sequence: reads binning before assembly, contig binning after assembly, and gene binning based on annotation (Alneberg et al., 2014; Cleary et al., 2015; Quince et al., 2017). Contig binning usually classifies contigs with similar composition and consistent abundance patterns into the same species. In addition, contigs binning has a better effect on contigs with higher abundance and is often applied by many software. It is possible to obtain low abundance species with reads binning, and even cluster species with abundance as low as 0.00001% (Cleary et al., 2015). Gene binning method is used in many published metagenome-wide association studies, and gene abundance is calculated (Wang and Jia, 2016). Currently, gene binning and contig binning are most widely used.

If the sequence assembly and sequence sorting are not correct, it is often impossible to identify new microorganisms. In the metagenomic sequencing report, species annotation and abundance statistics are required. The former refers to the analysis of species in the phylum, while the latter refers to the relative abundance of various microorganisms.

Metagenomic gene prediction is to use prediction tools to identify potential open reading frames (0RF) in genomic libraries and to predict the structure and function of unknown sequences of genes. Metagenomic gene prediction includes homology prediction and *de novo* prediction. Homology prediction can be obtained by alignment of homologous sequences with gene database, but the disadvantages are dependent on the existing gene database, and new genes cannot be annotated. *De novo* prediction is based on the sequence characteristics obtained by sequencing, which can predict both known and unknown genes based on different characteristics in the coding region and the non-coding region.

Function annotation is to compare a gene or protein sequence in a specific functional database, associate a gene or protein with a specific function, and help understand the relevant metabolic pathway. Through gene prediction and functional annotation analysis, the information of metabolic pathways can be obtained. To improve the quality of sequencing analysis, Bowers RM formulated Genome Sketch Standards of Metagenome-Assembled Genome (MAG) in 2017. High-quality drafts require gene integrity >90%, contamination <5%, and inclusion of 16S, 23S, and other highly conserved genes in assembly quantity (Bowers et al., 2017).

#### Commonly Used Software for Data Analysis of Sequencing Information

Data analysis relies on continuous improvement of existing databases and analysis software. Each process such as QC, binning, assembly, gene prediction, and function annotation has its own software with its own advantages. Megan is selected for the analysis and comparison of multiple groups of metagenome data, while the software GeneMark and metagenemark are often selected for gene prediction. In 2017, researchers compared and evaluated several software regarding assembly, binning and taxonomic profiling, to help researchers to select appropriate software (Sczyrba et al., 2017). The assembly software (MEGAHIT, Minia, and Meraga) performs well, assembling significant portions of the genome over a wide range and reducing misassembly. The MaxBin2.0 genome binner has the highest good performance (70–80% integrity, >92% purity), followed by other programs (MetaBAT, CONCOCT, and MetaWatt). The taxonomic software of PhyloPythiaS and Kraken have good results before the family level.

## Classification of Metagenomics

Metagenomics technology does not rely on isolation and culture but directly extracts the genetic material of microorganisms from the natural environment. Then, the characteristics of microbial communities can be studied through genetic analysis. Metagenomics is classified according to research purpose and research object.

### Metagenomics Can Be Divided Into Functional Metagenomics and Sequencing Metagenomics for Research Purposes

#### Functional Metagenomics Is Mainly Used for the Discovery of New Bioactive Substances and the Screening of New Microbial Functional Genes

Metagenomics technology has found many new genes, including the biocatalyst gene (Voget et al., 2003; Uchiyama et al., 2005), polyketide synthase coding gene (Ginolhac et al., 2004), antibiotic resistance genes (ARGs) (Riesenfeld et al., 2004), and others. In 2004, researchers used functional metagenomics to find that microorganisms in the soil had aminoglycoside and tetracycline antibiotic resistance genes that were significantly different from the original gene sequences in the database (Riesenfeld et al., 2004).

Active products produced by heterologous expression of metagenomic library genes, such as antibiotics, antitumor substances, and enzymes, are screened, sequenced, and analyzed. Metagenomics has great potential for application in the discovery of new enzymes without culture. Various enzymes found in metagenomes may be derived from microbial metabolism or microbial secondary metabolites. In the last two decades, metagenomics has shortened the discovery cycle of new enzymes, such as esterase, glycosidase, and oxido-reductase. Zhang et al. (2007) screened a specific low-temperature lipase at the South Pole. A strain showing extracellular lipolytic activity toward tributyrin was isolated from the deep-sea sediment of Prydz Bay and identified as a *Psychrobacter* species. The genomic DNA library was constructed from the strain with the highest lipolytic activity and the recombined plasmids were transformed into *E. coli*. The clones with lipase activity from 28,000 clones were identified. The insert DNA sequence of the selected clone was sequenced and an open reading frame of 954 bp coding was identified for a lipase gene by BLAST analysis. The effects of temperature on enzyme activity indicated that it was a typical cold-adapted enzyme.

There are many advantages to functional metagenomics. (1) Functional metagenomics can obtain target genes or active products through screening without culture conditions. (2) The development of new drugs was accelerated from marine and extreme environmental microbial resources. (3) Functional metagenomes help us to discover new members of existing enzyme families or enzymes that function only under specific physicochemical conditions, which can be better used in industry. (4) Functional metagenomics combined with metabolomics helped study the C, N, and S cycle metabolism of microorganisms in the environment.

There are many challenges in functional metagenomics. (1) The living conditions of the microbial community in the environment are complex, and the genes of the microorganisms with low abundance may not be extracted. (2) There is a loss of DNA fragments in the gene cloning process. (3) The frequency of heterologous expression of foreign genes is low. (4) The existing screening methods are limited and cannot meet all the enzyme screening requirements. (5) Screening is low efficiency, and only a few positive clones can be selected from tens of thousands of clones. Therefore, we need to develop higher throughput, high sensitivity, rapid, and simple screening methods in the future. (6) Due to temperature, pH, and other limitations, only a small part of the newly discovered enzymes can be used for industrial needs. (7) Current functional metagenomic studies are geographically uneven. It is mainly concentrated in developed countries. The vast microbial resources of developing countries are underexploited.

#### Sequencing Analysis of Metagenomics

To study the diversity and abundance of microbial community composition, species identification by microbial taxonomy is required. The traditional identification of microbial species composition is performed using the 16S/18S ribosomal DNA identification (16S/18S rDNA) in prokaryotic and eukaryotic cells, respectively; 16S rDNA sequencing is a commonly used molecular clock for bacterial taxonomic studies, but 16S rDNA provides limited information and may not be able to successfully identify species. Metagenomic sequencing is capable of identifying the species or the strain of a microbial community. For example, a research team used a combination of metagenome sequencing and single-cell sequencing to identify an extremely thermophilic bacterium *Candidatus kryptonia*, a new bacterium that could not be detected by 16S rDNA (Eloe-Fadrosh et al., 2016).

Metagenomic sequencing can be applied to bacteria, fungi, virus, and other microorganisms. In the absence of any available sequence information, metagenomic sequencing mainly uses *de novo* sequencing for microbial identification. Bioinformatics analysis methods were used to obtain the genomic sequence of microorganisms, such as COVID-19 (Wahba et al., 2020; Wu et al., 2020). In the presence of reference sequences, metagenome sequencing can focus on genetic variation information such as SNP, insertion/deletion of microbial significance, for example, the detection of YMDD (tyrosine—methionine—aspartic acid—aspartic acid) caused by point mutation of HBV polymerase gene region. At present, one of the hotspots of metagenomics research is disease-related human whole-exome sequencing (Wei et al., 2011).

There are many advantages to studying sequencing metagenomics. (1) Microorganisms in extreme environments are difficult to culture. Metagenomics can find such unculturable microorganisms. (2) Species, genetic, and evolutionary information of microorganisms can be obtained by studying the diversity of the microbial community. (3) New pathogenic microbial evidence can improve the current diagnosis of medical infections. (4) In terms of emerging infectious diseases, the target of pathogenic microorganisms can be quickly identified.

The sequencing of metagenomics is facing many challenges. (1) The DNA of environmental microorganisms cannot be extracted completely. (2) Sequencing process may miss low-abundance microorganisms. (3) There is no “gold standard” for sequencing processing software. (4) Because of the imperfection of the microbial database, many sequencing data might not be analyzed, including species annotation and functional analysis. (5) A sequencing platform with long reads and high accuracy is urgently needed. (6) The determination of the abundance of microorganisms in a microbial community is influenced by many factors.

### According to the Microorganism Studied, Metagenomics Can Be Divided Into Bacterial Metagenomics, Fungal Metagenomics, and Viral Metagenomics

At present, bacterial metagenomics is the most studied one, while the development of sequence analysis metagenomics is the fastest-growing one. Fungal and viral metagenomics are mainly used in sequence analysis metagenomics. Virus metagenomics can be used for systematic analysis and identification of overly scattered and low abundance viruses from the environment, a virus can be obtained directly from the environment without isolation and culture. With the advance in metagenome sequencing, most of the new information on virus databases comes from metagenomics rather than cultures. The Bundibugyo Ebola virus was discovered in 2007 in patients having Ugandan hemorrhagic fever using viral metagenomics (Towner et al., 2008).

## Application Fields of Microorganism Metagenomics

Applications of environmental metagenomics include the study of microorganisms in oceans, soils, deep oceans, glaciers, craters, and other environments. Metagenomics also has many applications in the medical field, such as the identification of pathogenic microorganisms in the intestinal tract, bloodstream infections, lung infections, central nervous system infections, and other infections. At present, metagenomic research focuses on antibiotic-resistant bacteria (ARB) and antibiotic-resistant genes (ARGs) (Gillespie et al., 2002; Riesenfeld et al., 2004; Arango-Argoty et al., 2018; Karkman et al., 2018; Nnadozie and Odume, 2019), biocatalysts, drugs, and others.

### Environmental Object of Microorganism Metagenomics Continues to Expand

Metagenomics is used in research related to agriculture, biology, pollution control, energy, environment, atmosphere, and other fields. Microbial subjects range from land to sea and extreme environments. There are huge microorganisms in the soil, and metagenomics studies have shown that soil is a potential cornucopia of antibiotics and antifungal agents. In 2002, the antibiotics Turbomycin A and Turbomycin B with broad-spectrum antibacterial activity were screened and named from the soil metagenomic library (Gillespie et al., 2002).

Compared with soil microorganisms, marine microorganisms have a broad prospect. Oceans, rivers, and lakes account for more than 70% of the earth's area, presenting great differences in the species, genes, and biological characteristics of microorganisms in the water. Marine microorganisms have special metabolites and metabolic regulation mechanisms. Metagenomic techniques are used to study the synthetic pathways of various marine secondary metabolites. A good example is marine actinomycetes-related metabolites (polyketone biosynthesis gene cluster), which can be used as a source of new drugs. In terms of ARGs, ARB levels in most river and lake systems are high, in particular, vancomycin-resistant *enterococcus* (VRE) and methicillin-resistant *Staphylococcus aureus* (MRSA) (Nnadozie and Odume, 2019).

The majority of extremophiles that survive in various types of extreme environments cannot be cultured artificially. In such extreme environments, it is more likely to obtain unique enzymes and abundant microbial resources. The active compounds can be screened by direct sequencing, sequence alignment with database, and functional analysis, some of which may be selected out for commercial development (Zhang et al., 2007).

### Metagenomics for Prevention and Control of Environmental Pollution

Bioremediation is a new bioengineering technology that uses the natural purification ability of environmental microorganisms. Bioremediation is expected to reduce pollutants or make them harmless by using the metabolic activities of microorganisms in the treatment system. Bioremediation needs several conditions: (1) Pollutants are organic and can be degraded by microorganisms. (2) The microorganisms possess certain metabolic activities. (3) The environment is suitable for degradation, meanwhile, for the maintenance of microbial activity. Metagenomics may help explore new functional microorganisms and genes, and construct and screen out new microbial strains with high degradation efficiency, broad applicability, and stable expression.

In the sewage treatment project, not only the organic matter in the sewage needs to be degraded but also the nitrogen and phosphorus elements in the sewage need to be removed. The wastewater treatment process deals with diverse microbial populations, such as anaerobic ammonium oxidation bacteria, phosphorus-accumulating bacteria, and electrochemically active bacteria. Metagenomic studies of water microorganisms contribute to biological nitrogen removal processes and enhanced biological phosphorus removal. Functional genes have been found in microorganisms that can degrade pesticides, plastics, polycyclic aromatic hydrocarbons, petroleum hydrocarbons, and other organic pollutants, which can play an important role in environmental bioremediation. Researchers screened a variety of new proteolytic enzymes from yard compost to degrade pesticides (Lämmle et al., 2007). In 2005, a researcher screened benzoate anaerobic degradation genes from Black Sea sediment, which contributes to the anaerobic degradation of aromatic compounds (Krüger et al., 2003). Microbial electrochemical systems (MES) use electroactive bacteria enriched in anodes to recover electrical energy directly from organic waste (Wang and Ren, 2013; Venkata Mohan et al., 2014). As an emerging sewage treatment technology, MES has been developed rapidly.

### Metagenomics Are Used in the Medical Diagnosis of Pathogenic Microorganisms

Metagenomics findings suggest that the intestinal microbiome is associated with a variety of diseases, such as non-alcoholic fatty liver disease (Aron-Wisnewsky et al., 2020), autoimmune diseases (Svoboda, 2021), and tumors (Andrews et al., 2021). Metagenomics can be used to detect drug-resistant genes for pathogens and monitor outbreaks of infectious diseases in hospitals and communities. In 2021, the Laboratory Medicine Branch of the Chinese Medical Association published an expert consensus, recommending the use of NGS on the samples of suspected infection sites in case that no evidence of etiology is obtained by routine biochemical examination or microbial culture, and empirical anti-infection therapy is ineffective. It is recommended to select sequencing metagenomics when the cause and site of infection are unknown and the pathogenic microorganisms cannot be determined by the existing detection technology. NGS can deal with a wide range of pathogenic microorganisms, including bacteria, viruses, fungi, and parasites. Samples may be collected in case of suspected central nervous system infection, respiratory tract infection, bloodstream infection, etc. Now metagenomic sequencing has been widely used in the detection of medical pathogens. Respiratory RNA viruses such as Picornaviridae, Coronaviridae, Paramyxoviridae, and Orthomyxoviridae can be diagnosed by NGS (Thorburn et al., 2015). Five organisms (*Neisseria meningitidis, Streptococcus agalactiae, Candida albicans, Mycobacterium fortuitum*, and *Mycobacterium abscessus*) were detected by mNGS testing in the cerebrospinal fluid (Miller et al., 2019).

Limitations of metagenomics in the medical diagnosis of pathogenic microorganisms are as follows: (1) Pathogenic microorganisms detected by metagenomics might be dead or dormant microorganisms, and their clinical significance should be carefully judged by combining with other clinical data. (2) Real-time quantitative PCR has been applied in clinical practice, and its stability and accuracy have been confirmed. While the accuracy of metagenomics on microbial abundance needs to be verified by large sample data. (3) In the case of intracellular bacterium infection, the false-negative result may occur due to an incorrect cell wall rupture method. (4) It is recommended that metagenomics should not be the first choice when immunoassay or PCR methods are available for pathogen diagnosis. (5) When interpreting whether the SNP in microbial genes is related to function, large-scale clinical trials are needed to verify the conclusion. (6) It is important to remove DNA contamination from environmental microorganisms or hosts before data analysis.

### Applications of Metagenomics in Other Aspects

Metagenomics studies the diversity, species composition, and genetic evolution of microorganisms in different environments. People have applied metagenomics to winemaking, farming, and authentication of Chinese medicine. It is found that the diversity of microbial composition and population characteristics in a wine cellar is very important to improve the flavor and quality of the wine (Stefanini and Cavalieri, 2018; Liu et al., 2021). The pharmaceutical value of Chinese medicine from different producing areas is different, which may be related to the composition of microorganisms under their unique climate and soil. The increase in crop yield is inseparable from the correlation between plant roots and soil microbial community (Sharma et al., 2018).

## Outlook

At present, metagenomics is advancing rapidly in many fields. The bottlenecks of functional metagenomics lie in the failure of large fragment DNA extraction and expression of genes from metagenome libraries in heterologous hosts. High-throughput screening in metagenomic libraries is also a problem to be solved in the future. The development of NGS and TGS technology has promoted the progress of metagenomics. Although the cost of NGS sequencing is reducing and the speed of sequencing is increasing, the microbiological database is incomplete, so it is difficult to correctly assemble and bin without sequencing data for reference. For example, most viral sequences obtained by viral metagenomics have no similarity to any known sequences in the existing databases and, therefore, cannot be annotated for gene and function. In the future, with the progress of TGS sequencing technology, the accuracy rate will be greatly improved. The emergence of new computer algorithms can better process post-sequencing data.

In the future, metagenomics will be combined with metatranscriptomics, proteomics, and metabolomics. Metagenomics provides genetic information of all the environmental microorganisms. Metatranscriptomics is to study the species, functions, and expression activities of transcripts in a microbial community at the RNA level. Metaproteomics can also provide real-time information on the functional expression of environmental genes, while metabolomics studies the metabolic pathways of metabolites. The linking of these omics will lead us to study from genes to proteins and from structure to function.

## Author Contributions

YX and ML contributed to the study concept and design. LZ, FC, ZZ, MX, FS, LY, XB, YL, YG, and HH collected and sorted out the literature. LY, YX, and ML drew pictures. LZ, FC, and ZZ wrote the first draft. WY, ML, and YX edited and approved the English version of the article. All authors contributed to the article and approved the submitted version.

## Funding

This research was supported by the Beijing Hospitals Authority Clinical Medicine Development of Special Funding Support (Nos. XMLX 201706 and XMLX 202127), the Special Public Health Project for Health Development in Capital (2021-1G-4061), the Digestive Medical Coordinated Development Center of Beijing Hospitals Authority (Nos. XXZ0302 and XXT28), the National Science and Technology Major Project of China (Nos. 2017ZX10201201-001-006 and 2017ZX10201201-002-006, and 2018ZX10715-005-003-005), the Beijing Municipal Science & Technology Commission (No. Z151100004015122), the Beijing Science and Technology Commission (No. D161100002716002), and the National Key R & D Projects No. 2020YFC0846200.

## Conflict of Interest

The authors declare that the research was conducted in the absence of any commercial or financial relationships that could be construed as a potential conflict of interest.

## Publisher's Note

All claims expressed in this article are solely those of the authors and do not necessarily represent those of their affiliated organizations, or those of the publisher, the editors and the reviewers. Any product that may be evaluated in this article, or claim that may be made by its manufacturer, is not guaranteed or endorsed by the publisher.
